# Hip Joint Disease

**Published:** 1891-05

**Authors:** P. H. Fitzhugh


### "HIP JOINT DISEASE."

By P. H. FITZHUGH, M. D.

*Concluded.*

### DIFFERENTIAL DIAGNOSIS.

The affections most frequently mistaken for hip disease are: 1, Synovitis; 2, Lumbar, Pott's; 3, Neuroses of the Hip; 4, Infantile Paralysis; 5, Peri-Articular Disease; 6, Peri-Nephritis and Peri-Typhlitis; 7, Congenital Dislocations.

The symptoms of beginning hip disease and those of synovitis may be so similar that a diagnosis at first is impossible. Synovitis may begin suddenly without known cause, or following a fall, with extreme pain, fever, immobility and swelling. These symptoms may soon subside and complete recovery of the joint follow; or the subsidence may be more gradual, the symytoms lasting for months. All of the usual joint symptoms, such as muscular spasm, etc., may be present in chronic forms of synovitis. In adults synovitis comes on most frequently after a fall, or traumatism of some kind. It is much more common in adults than in children. Where recovery takes place in a few months or less, in cases marked by characteristic hip disease symptoms, it may be inferred that ostitis never existed, but that it was purely a synovitis.

Lumbar, Pott's disease, may cause a limp and restriction of motion of one hip, thereby simulating hip disease so closely that it may be very difficult to diagnose. As a rule the motion is only limited in one direction, viz., hyper-extension; the contraction of the psoas producing this result may be caused by the descent of pus into the psoas or to irritation reflex to the disease. Where an abscess burrows near the joint it may so irritate the joint that all of the motions will be restricted. This, however, where the joint is not involved, soon passes off under proper rest, with the exception of restriction to hyper-extension or extension. Abduction, which is limited very early in hip disease, most often remains free in even well marked cases of psoas contraction. Hysterical hip may resemble true hip disease in many particulars. Excessive pain may be complained of, especially on manipulation, but the pain is more apt to be *referred* to the *hip than the knee;* while the *reverse holds good* in *true hip disease.*

Atrophy may or may not be present.

On examination the limb is usually held very stiff, unless the attention of the patient can be withdrawn, when motion will be greater. I remember examining a woman who presented herself at the hospital for double hip disease. She walked very poorly, allowing only the slightest amount of motion at the hip joints. On examination in the recumbent position, we found all motions limited; neither thigh could be flexed to an angle less than 160°, but the patient, on being told to get up, flexed the thigh to about 90° which confirmed beyond a doubt the diagnosis of hysterical hip.

Congenital dislocation can hardly be mistaken for hip disease; the limp being the only symptom, and this having existed since the child commenced walking. In hip disease the limp is not congenital. There is no limitation of motion, muscular spasm being entirely absent.

INFANTILE PARALYSIS.--In occasional cases there may be at the beginning of the paralysis " extreme pain and tenderness with immobility of one limb ; ordinarily these symptoms are not accompanied by other symptoms of hip disease," and are followed by loss of power and warmth of the affected limb, atrophy of the whole limb rapidly taking place.

Peri-articular disease may be recognized from the inflammation attacking certain groups of muscles, causing a limitation to motion greater in one direction than another, " while in true joint disease the limitation is equal in all directions of the normal motion of the joint."—(Bradford and Lovett.)

Peri-nephritis and peri-typhlitis have been mistaken for hip disease. These affections may present psoas contraction with or without iliac abscess. The limitation to motion is not general, affecting principally extension; abduction, etc., remaining quite free.

PROGNOSIS.

Under favorable circumstances the tendency of hip disease is to recovery, with more or less deformity, the amount of deformity depending largely upon the mode of treatment and the care taken of the patient. Cavin reported eighty cases of suppurative hip disease treated at the hospital at Berck ; in the course of five years 50 per cent. were cured ; 12½ per cent. died ; 25 per cent. were not cured; 7½ per cent. were improved when removed.

"In fifteen cases of suppurative coxitis with albuminuria, five died under conservative treatment ; two were discharged improved; six not improved; two cured. These cases of Cavin's were, with the exception of ten, severe cases sent from the Paris hospitals after they had ceased to improve there." Gibney, from an analysis of 288 cases, reported a mortality of 12½ per cent. from exhaustion, meningitis and amyloid degeneration. " Shaffer and Lovett investigated fifty cases of cured hip disease which had been discharged from the New York Orthopedic Dispensary at least four years previously, and found that forty-one had remained cured. Of the remaining ten, four had died, and six had relapsed, although four of the latter had been apparently cured a second time."

In ninety-six deaths after suppurative hip disease at the Alexandria Hospital, London, the causes were as follows :

| | |
|---|---|
| Meningitis, | 16.7 |
| Albuminuria from dropsy, | 20.8 |
| Phthisis, | 5.2 |
| " and albuminuria, | 3.1 |
| Exhaustion, | 9.4 |
| Erysipelas and pyæmia, | 3.1 |
| Intercurrent disease, | 7.3 |
| After operation, | 9.4 |
| Unknown, | 25.0 |

In fifty-one cases investigated at the New York Orthopedic Dispensary and Hospital, treated by purely conservative means, fifty-one cases were discharged cured. Forty-one of these remained well and were able to do a full day's work at their respective occupations; only one case, and that combined with Pott's disease, used a cane ; none used crutches. Among these were printers, mechanics, errand boys, shop girls, school children, etc., and none presented any evidence of active tubercular disease, or serious incapacity from deformity. Dr. Gibney, in 1878, reported eighty cases cured at the hospital for Ruptured and Crippled, New York, by expectant treatment and internal medication. Forty-eight developed abscesses ; in the remaining thirty-two, none had abscess. Thirty-three of these cases ran their course in three years, twenty-eight in from three to six years, and in nineteen cases from six to ten years. At the termination of the disease, sixty-one of these patients could walk and run without discomfort; twelve walked fairly, at times requiring a support, and seven could walk only with the aid of crutches. In twelve of these cases an arc of at least 15° of motion existed in the afflicted joint. From one to three inches represented in the majority of cases the amount of shortening.

In few diseases is the good effect of thorough, skilful treatment, more evident; but it should be continued for some time after all signs of the disease shall have disappeared.

The average length of time required for treatment is about 2½ to 3 years, but it is better to continue the treatment six months or a year longer, than to remove the splint one week too soon, for extension and immobilization of the joint for a year longer could produce no damage, whereas the change to a convalescent splint too soon may produce a severe relapse. The presence or absence of abscess does not seem to affect the outlook in regard to motion. In hip disease, where proper and skilful treatment is commenced at an early stage and continued for some time after all symptoms have ceased, the prognosis as to the position and function of the limb is favorable; but, should recovery take place with the limb ankylosed in a distorted position, such as marked flexion, abduction or adduction, the deformity can be entirely overcome, and a useful limb restored by means of operative interference. The real shortening existing will be permanent, but this, unless great, will not be of much moment, as tilting of the pelvis will readily assist in equalizing the length, or the shoe of that foot may be built up.

The atrophy also is claimed never to be entirely cured, but as soon as the patient removes the splint and begins to use the limb, the atrophy diminishes very much.

### TREATMENT.

The object in the treatment of hip disease should be to afford protection, fixation and extension to the diseased joint; to prevent deformity, build up the general condition, and at the same time keep a lookout for and be prepared to meet any and all complication, such as abscess which may arise. I will not describe many of the different modes and appliances for the treatment of coxalgia, but will only mention the principal ones in daily use, at the hospital for Ruptured and Crippled, New York. For fixation are used: 1, plaster Paris; 2, wire cuirass; 3. Thomas hip splint; and 4, De Pass's modification of the same. The plaster of Paris, as ordinarily applied, does not produce the desired amount of fixation, from the fact that it does not grasp the trunk firmly enough to prevent motion in the lumbar vertebræ. This permits the pelvis to move within the bandage, allowing motion at the hip. While this objection is very vigorously urged by many orthopedists, still the writer is convinced that it is largely and scientifically exaggerated. In the writer's opinion it can be so applied as to produce as fine fixation as any purely fixation splint known to him. Prepare the patient as follows: First, grease with vaseline—I am aware that this step is objected to by some, but they are largely in the minority—hold the limb in as good position as possible, pad well the bony prominences and lower ribs, then run a spica bandage of thin cloth from the toes to and including part of thorax; over this apply a light, smooth plaster Paris bandage, being very careful to remove all "bricks," or lumps of plaster Paris, and have each layer perfectly smooth, so that there will be nothing liable to excoriate.

A plaster Paris bandage applied to the naked skin, nicely and smoothly, need give no alarm in regard to excoriations. It is the roughly applied, lumpy bandage, one layer being tighter than the other, which causes the trouble. Increase the strength by a light bar of steel running over the groin, and by reinforcing with the bandage at this and other weak points. By this means, we have a light, firm and solid support from the malleoli to very near the axillæ. This having been applied without much padding must necessarily hold the part pretty firm. It certainly affords the greatest amount of relief to the most exquisitely sensitive joint. I have frequently seen children, crying from intense pain, who were relieved a short time after this application, and remained so until after its removal.

The "wire cuirass" furnishes very good fixation, but it is quite expensive, and more uncomfortable than the plaster Paris, and is not at all fitted for dispensary work.

Thomas' fixation hip splint, invented by Hugh Owen Thomas, of Liverpool, is largely used, both in this country and in England. It is a very useful splint, and has many redeeming points. It is very simple of construction, light in weight, and quite firm and secure when properly adjusted in its object of fixation. It consists of an iron bar extending from the inferior angle of the scapula to the lower third of the posterior surface of the leg; to the upper end is attached a band encircling to the waist at right angles to the upright iron bar. Just below the gluteal fold, and at the lower end of the upright bar are placed other bands at right angles encircling about two-thirds of the thigh and calf respectively. This is held in position by a canvass lacing around the trunk and bandage to the limbs. It can be so bent as to fit any angle of deformity, and gradually straghtened as the spasm of the muscles wears off. Mr. Thomas claims that by this means he can afford protection and fixation to the joint, and at the same time correct any existing deformity, *i. e.*, where it is not due to bony ankylosis.

While this splint has given remarkable satisfaction in Mr. Thomas' hands, it has not done so well by others, and we are convinced that fixation can hardly be made absolute.

My friend, Dr. Matt De Pass, of the hospital for Ruptured and Crippled, New York, has so modified the Thomas hip splint as to vastly increase its usefulness. He extended the length of the posterior iron bar to two or three inches below the sole of the foot, when it bent at right angles upon itself; to this bent piece is attached a cross bar with straps at each end; these are fastened to buckles in adhesive plasters applied to the leg. By this extension can be produced, counter-extension being obtained by two perineal straps fastened to an additional band encircling the pelvis at right angles, and attached to the posterior bar.

The writer remembers two girls with very acute "hips" on whom had been tried the Thomas splint without any amelioration whatever; in fact they were apparently daily growing worse from inability to keep the splint perfectly adjusted. On these two Dr. De Pass applied his modification. For some time previous they had been averaging from twenty to thirty-five "night cries" every night. The night following the application of the modified splint the cries were reduced about 50 per cent.; on the second night one was reported as having cried eight times, the other less. The improvement was not merely temporary, but permanent.

On the sound foot a 3½ to 4½ "Patton" is applied, the patient using crutches. This prevents any standing on or jarring of the diseased limb. This "Patton" is used in connection with all

Thomas traction splints. The objection to all of the purely fixation splints is, that they do not prevent the muscular spasm from crowding the head of the femur into the acetabulum. This spasm prevents rest of the joint, increases the existing inflammation of the bone, and permits destruction of the femur and enlargement of the acetabulum, thereby contributing to the distortion of the limb.

The traction splints used are known as the "Taylor Hip Splint," or Gibney's modification, the "Polyclinic Hip Splint." The virtue of these, as in fact all traction splints, depends upon practically the same principle, *i. e.*, traction on the limb with counter-traction or resistance at the perineum. Taylor's splint consists of a pelvic girdle ; two perineal straps and a long outside bar extending from the trochanter to any desired length below the foot, the upper end of this bar is attached firmly by a screw to the pelvic girdle, where it passes over the trochanter. The lower end is broadened and bent upon itself, so as to pass under the foot at right angles. To this broadened foot piece is attached two leather straps which can be fastened into buckles secured to the adhesive plaster on the patient's leg. The stem consists of two pieces ; one "hollow at the lower part, with teeth cut on the edge into which a rod plays, by means of a key." The rod can be moved up and down, and it is caught and held in place by means of a spring and sliding catch."

Gibney's modification, known as the "Polyclinic Hip Splint," is similar to the above, with the exception of the stem being made of one piece. To the foot piece of this stem may be attached a windlass with a ratchet, to which the traction straps are fastened. By turning the key to the windlass traction to any desired extent may be made. Unless great care is taken the perineum is very liable to become excoriated. It should be bathed with 50 per cent. alcohol frequently, and dusted daily with some dry impalpable powder, such as talc, subiodide of bismuth or zinc oxide. A most excellent application, one that I recommend from personal experience, is

℞. Pulv. amyli., ʒvi.

Pulv. zinci oxidi, ʒiss.

Pulv. camphoræ, ʒss.

M. Sig.—Dusting powder.

The leg is quite apt to excoriate, hence the plaster should be removed on an average of every two weeks.

The excoriated leg should be treated as the perineum, or an ointment, such as the following may be applied :

℞. Acid salicylic, gr. xv.

Pulv. zinci oxidi,

Pulv. amyli., aa. ʒii.

Vaseline, q. s. ℥i.

M.

The weight and pulley are a valuable addition in the treatment of malpositions due to muscular spasm. The patient being put on a "cabbot frame" with an incline and traction made in the line of deformity, which is corrected daily as the spasm decreases. Where the deformity cannot be corrected by the weight and pulley it may be necessary to resort to surgical procedures, such as: 1, Manual force under ether; 2, tenotomy, myotomy and fasciotomy; 3, osteotomy.

"Bessement force" under ether is often all that is necessary. At all times it is worth the trial before more radical procedures are resorted to. The objections to this are the liability to fracture when an undue amount of force is used, and the possibility of re-establishing the disease from the setting free of an encapsulated focus of tuberculosis material, which had become inactive. Myotomy, etc., should be resorted to when the above procedure is not sufficient to correct the deformity, and you can feel the tense bands holding the limb. Cut subcutaneously and divide carefully all constricting bands. One can hardly cut too much, provided he avoids the larger vessels and nerves.

When bony ankylosis has occurred subtrochanteric osteotomy, known as "Gant's operation," is by far the most preferable. Mr. Gant, in the *British Medical and Surgical Journal* of October 18th, 1879, states the following as the reasons why his operation is to be preferred: "When, in consequence of continued disease of the hip-joint, the head of the femur has disappeared, leaving only a stunted nodule of bone representing the neck above the trochanter, in such a case the operation of section in the femoral neck cannot be performed, there being no neck to divide. Even when supra trochanteric section is practicable, the state of the neck may render the operation abortive. The seat of the operation will be in an almost carious portion of the bone which is unfit to yield a fibrous union." In Gant's operation the psoas and iliacus, acting above the division, are not so apt to reproduce flexion as in the older operations, where they acted below the point of section.

Provided with a chisel and mallet, the patient lying on the sound side with a sand bag between the thighs, the skin having been thoroughly scrubbed and washed with bichloride, sol. 1 to 3000. About an inch or an inch and a half below the trochanter major, push the chisel with the blade in the long axis of thigh, down to the bone, through the periosteum, when it is turned until its edge is at right angles to the long axis of the femur. The chisel or osteotome should then be driven by sharp blows with the mallet first forward then backward until the shaft has been cut about three-quarters through, when an assistant grasps the pelvis, the operator attempting to complete the division by fracture. Should this not be possible, repeat the chiselling until it is. The bone breaks with a loud snap and can generally be brought into the divided position without further trouble. Oil silk is then applied over the incision; around this, bichloride gauze and cotton, then a plaster of Paris spica. This is allowed to remain about four weeks, unless indications to the contrary should prevent. When it is removed and the position and condition of the limb noted, another plaster of Paris is applied for about two weeks longer, an ambulatory splint being then substituted.

Simple uncomplicated abscesses need cause no alarm. They may be left alone or aspirated. Should it become extensive and painful, free incision, thorough scraping of the sac and diseased focus, irrigation with bichloride, 1 to 4000, and dusting with iodoform should be resorted to. The incision may then be closed with silk or left to close by granulation. Salicylate of soda, antifebrin, opium, chloral, and the bromides have all been used with varying degrees of success, for " night cries." The main objection to their use is the injurious effect of the narcotics upon the appetite, digestion and nervous system. The most efficient means are fixation, with extension made in the line of deformity.

No general rule can be laid down for the proper treatment, each individual case being a study within itself. In one case firm fixation with rest in bed for some time may be deemed advisable, owing to the sensitiveness of the joint or activity of the ostitis, while in other cases ambulatory appliances from beginning to end will be all that is necessary. As the *pain* and *muscular spasm* pass off, the traction may be suspended and the joint merely protected from jar as long as there is any possible tendency to a recurrence of the muscular spasm. By a proper appreciation of the symptoms, meeting all as they present, and the continuance of the treatment very carefully, for at least a year or two, with a vigilant supervision and protection for several years subsequently, we should expect the diseased limb to recover without distortion and with considerable if not almost perfect motion.

